# *Sargassum horneri* extract fermented by *Lactiplantibacillus pentosus* SH803 mediates adipocyte metabolism in 3T3-L1 preadipocytes by regulating oxidative damage and inflammation

**DOI:** 10.1038/s41598-024-65956-8

**Published:** 2024-07-02

**Authors:** Jae-Young Kim, Sejin Jang, Hyun Ji Song, SangHoon Lee, Sejin Cheon, Eun Jin Seo, Yi Hyun Choi, Sae Hun Kim

**Affiliations:** 1 College of Life Science and Biotechnology East Building, Korea University, 145, Anam-ro, Seongbuk-gu, Seoul, 02841 Republic of Korea; 2 Institute of Life Science and Natural Resources, Korea University, Seoul, 02841 Republic of Korea

**Keywords:** *Sargassum horneri*, Fermentation, *Lactiplantibacillus pentosus*, Oxidative stress, Inflammation, Adipogenesis, Biotechnology, Microbiology

## Abstract

*Sargassum horneri* (*S. horneri*), a brown seaweed excessively proliferating along Asian coastlines, are damaging marine ecosystems. Thus, this study aimed to enhance nutritional value of *S. horneri* through lactic acid bacteria fermentation to increase *S. horneri* utilization as a functional food supplement, and consequently resolve coastal *S. horneri* accumulation. *S. horneri* supplemented fermentation was most effective with *Lactiplantibacillus pentosus* SH803, thus this product (F-SHWE) was used for further in vitro studies. F-SHWE normalized expressions of oxidative stress related genes *NF-κB*, *p53*, *BAX*, *cytochrome C*, *caspase 9*, and *caspase 3*, while non-fermented *S. horneri* (SHWE) did not, in a H_2_O_2_-induced HT-29 cell model. Moreover, in an LPS-induced HT-29 cell model, F-SHWE repaired expressions of inflammation marker genes *ZO1, IL1β, IFNγ* more effectively than SHWE. For further functional assessment, F-SHWE was also treated in 3T3-L1 adipocytes. As a result, F-SHWE decreased lipid accumulation, along with gene expression of adipogenesis markers *PPARγ*, *C/EBPα*, *C/EBPβ, aP2*, and *Lpl*; lipogenesis markers *Lep, Akt, SREBP1, Acc, Fas*; inflammation markers *IFN-γ* and *NF-κB.* Notably, gene expression of *C/EBPβ, IFN-γ* and *NF-κB* were suppressed only by F-SHWE, suggesting the enhancing effect of fermentation on obesity-related properties. Compositional analysis attributed the protective effects of F-SHWE to acetate, an organic acid significantly higher in F-SHWE than SHWE. Therefore, F-SHWE is a novel potential anti-obesity agent, providing a strategy to reduce excess *S. horneri* populations along marine ecosystems.

## Introduction

*Sargassum horneri* is a brown seaweed found along the coast of China, Korea, and Japan^1^. Recent climate changes and coastal eutrophication have contributed to the proliferation of *S. horneri* population, leading to a massive influx along the Korean coast. Coastal *S. horneri* has been used as fertilizers; however, the excess *S. horneri* beyond the demand of local farms is difficult to handle. To address this, scientists have been exploring ways to utilize *S. horneri*, a vitamin, flavonoid, amino acid, polyphenol, and polysaccharide-rich bioactive substance, as a cosmetic or food material^2^. Due to its rough texture, *S. horneri* has been cosmetically used as a face mask; however, its application in food is still limited^3^. Interestingly, *S. horneri* oral supplementation, along with a high-fat diet, suppressed fat accumulation in adipose tissue and liver^4^. Therefore, studies on using *S. horneri* as a food additive instead of a standalone food ingredient are in progress. In a previous report, supplementation of *S. horneri* enhanced lactic acid bacteria (LAB) growth, revealing its potential as a prebiotic supplement^1^. This suggests that fermentation could be utilized to enhance *S. horneri* biological functions through production of secondary metabolites. LAB are gram-positive, lactic acid-producing organisms, in which possess numerous health benefits. Major strains include probiotics—live microorganisms that confer health benefits to the host when administered in adequate amounts^5^. Among probiotics, strains from the *Lactobacillaceae* and *Bifidobacteriaceae* families are involved in fermentation; they regulate microbial growth and the enzymatic conversion of food constituents^6,7^, which in turn produces beneficial end-products, such as organic acids, bacteriocins, and peptides^8^. In a previous report, *S. horneri* fermentation with *Lactiplantibacillus pentosus* SN001 increased glycerol levels, in which elevated antihypertensive effects^9^. Therefore, fermentation is a prominent tool for enhancing the value of biomaterials.

Obesity, the state of excess fat accumulation, is a global crisis affecting more than 1 billion people, including 650 million adults, 340 million adolescents, and 39 million children^10^. Obesity-inducing energy overconsumption contributes to symptoms such as adipogenesis, increased fat cell numbers, and adipocyte hypertrophy^11^. Adipogenesis, or adipocyte hyperplasia, is the process of adipocyte precursor cells committing their fate to the adipogenic lineage, gathering nutrients, and transforming into triglyceride-filled mature adipocytes^11^. This determines the lipid storage capacity of adipose tissue; therefore, it is a potential therapeutic target for treating obesity. Furthermore, obesity-associated fat accumulation induces inflammation and hypoxia, which increases oxidative stress^12^. Oxidative stress in turn exacerbates inflammation, leading to obesity-related complications, such as type 2 diabetes, insulin resistance, and infertility^13^. *S. horneri* and LAB possess antioxidative and anti-inflammatory properties, which suggests their potential in attenuating obesity^14–16^. Moreover, in previous reports, the antioxidative activities of *Sargassum* spp. were enhanced by LAB fermentation^10,17^. Therefore, this study aimed to determine the anti-obesity potential of *S. horneri* and LAB combined through fermentation in vitro. Our study introduces a novel perspective on the utilization of *S. horneri* as an edible source, which has not been frequently addressed due to its rough texture. Moreover, our study aims to enhance functional properties of *S. horneri* through fermentation, and ultimately heighten the value of *S. horneri* as a food product.

## Results

### Selection of the optimal *S. horneri* fermentation product and assessment of its antioxidative activity

**Figure 1:**
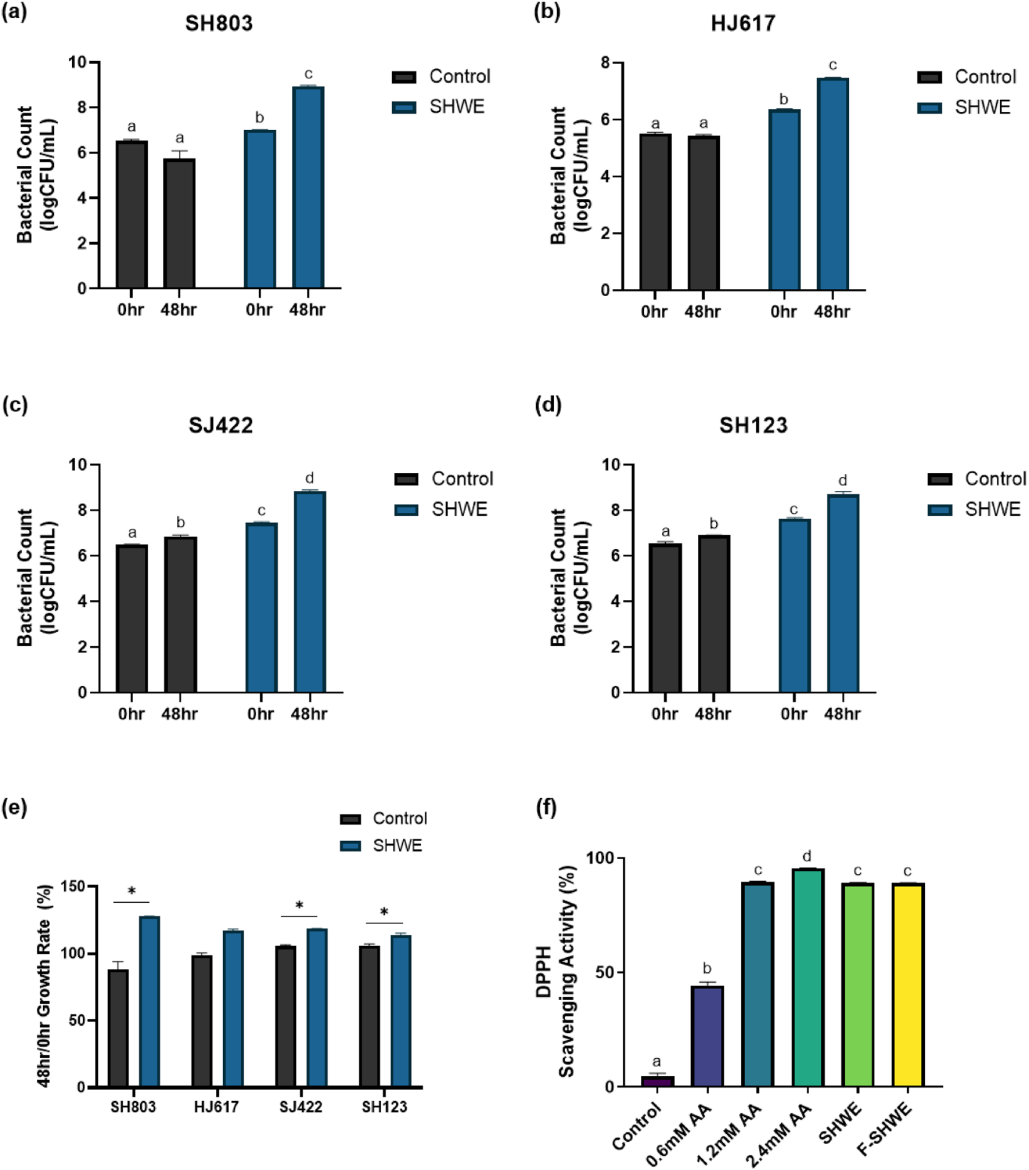
Screening of *S. horneri*-fermentable bacteria and evaluation of antioxidative properties of the selected product (F-SHWE). (**a**–**d**) Bacterial growth of lactic acid bacteria (LAB) strains cultivated in growth media or growth media supplemented with 2% SHWE for 48 h. Results are expressed as mean ± standard error (SE) (*n* = 3). ^abc^Results in the same series with different lowercase superscript letters are significantly different (*p* < 0.05). (**e**) Growth rate of LAB strains cultivated in growth media or growth media supplemented with 2% SHWE for 48 h. Results are expressed as mean ± SE (*n* = 3) (*p < 0.05, **p < 0.01, ***p < 0.001, compared with the control group) (**f**) DPPH scavenging activity of SH803 fermented SHWE (F-SHWE). Results are expressed as mean ± SE (*n* = 3). ^abcd^Results in the same series with different lowercase superscript letters are significantly different (*p* < 0.05).

Combinations of SHWE with bacterial strains were screened to identify the bacterial strains capable of using *S. horneri* as an energy source (Fig. 1a–e). SHWE supplementation in bacterial growth medium did not inhibit the growth of all four bacterial strains at 48 h, indicating non-toxicity (Fig. 1a–d) (*p* < 0.05). Moreover, the 48 h growth rates of SH803, SJ422, and SH123 significantly increased through SHWE supplementation (Fig. 1e) (*p* < 0.05). Specifically, the growth rate of SH803 for 48 h compared to the 0 h in the control medium was 87.98%, which increased to 127.68% with SHWE supplementation. Not only did SHWE supplementation enhance the 48 h growth rate compared to the 0 h by 39.7% in SH803, but also 12.95% in SJ422, and 8.36% in SH123. Based on these results, SHWE was the most effective in improving SH803 proliferation. Therefore, SH803 F-SHWE was used for further assessments. In vitro DPPH radical scavenging activity was measured to determine the antioxidative potential of F-SHWE (Fig. 1f). SHWE and F-SHWE showed high DPPH radical scavenging activities equivalent to 1.2 mM ascorbic acid (*p* < 0.05). However, the antioxidative activity of the non-supplemented growth media was 4.72%, which was significantly lower than that of SHWE or F-SHWE (89%, *p* < 0.05). Thus, SHWE enhanced the antioxidative effects, and fermentation did not suppress these properties.

### Effects of F-SHWE on H_2_O_2_-induced oxidative stress in HT-29 intestinal epithelial cells

**Figure 2:**
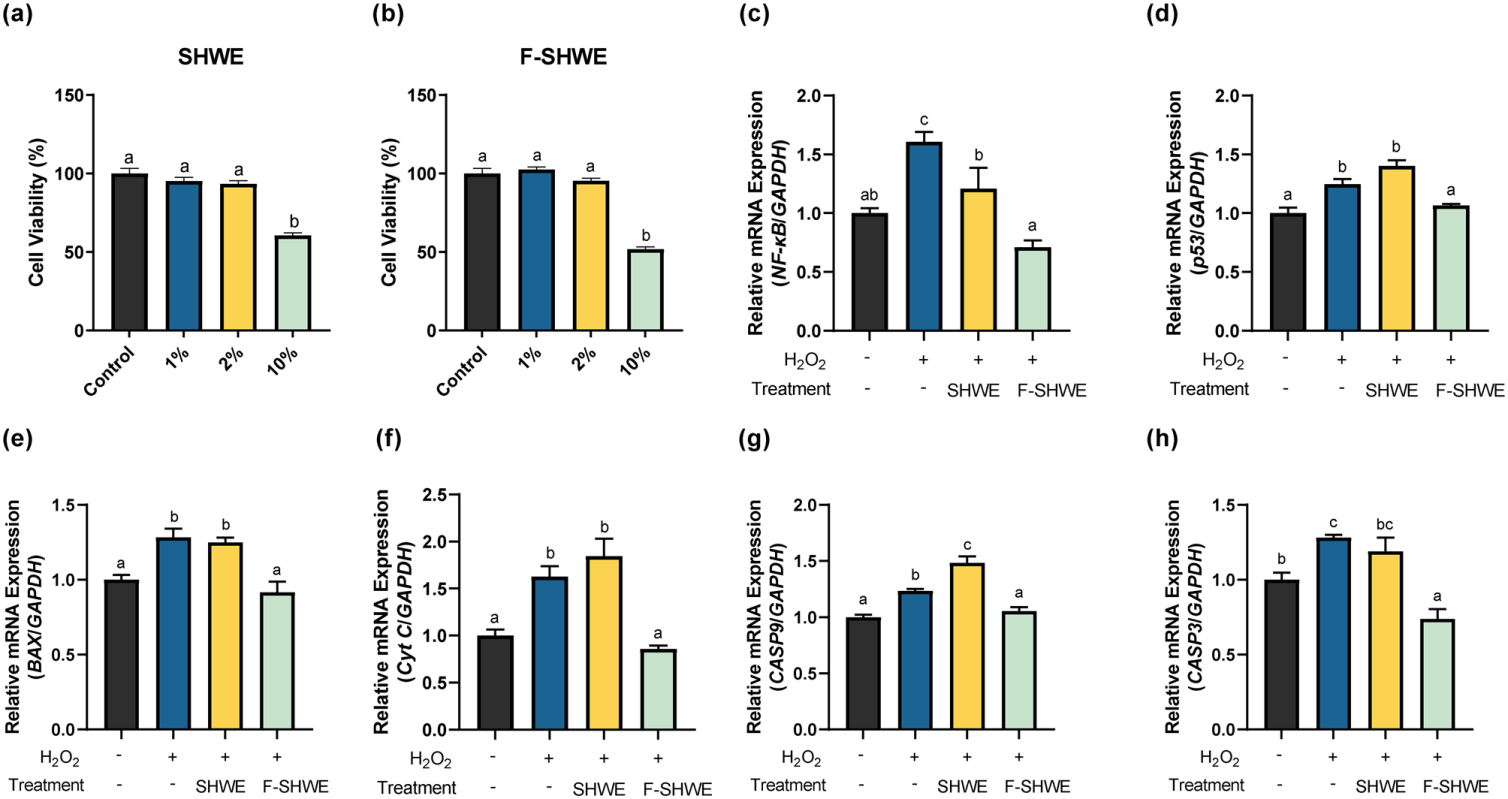
Effects of F-SHWE on H_2_O_2_-induced oxidative stress in HT-29 cells. (**a**,**b**) Viability of HT-29 cells pretreated with different concentrations (0, 1, 2, 10%) of SHWE or F-SHWE for 24 h. (**c**–**h**) Effects of SHWE or F-SHWE pre-treatment on the gene expression of apoptosis-related markers in H_2_O_2_-treated HT-29 cells. Results are expressed as mean ± SE (*n* = 3). ^abc^Results in the same series with different lowercase superscript letters are significantly different (*p* < 0.05; *Cyt C*: *Cytochrome C, CASP9*: *Caspase 9, CASP3*: *Caspase 3*).

The antioxidative properties of F-SHWE were further evaluated using an oxidative stress-induced cell model. The optimal dosage for in vitro assays was selected based on cell viability measurements using the MTT assay (Fig. 2a–b). SHWE or F-SHWE treatments reduced cell viability to 51.79% at a dose of 10% (*p* < 0.05). Lower doses of SHWE and F-SHWE (1–2%) maintained cell viability at 90% (*p* < 0.05). As cell viability reduction > 30% is considered cytotoxic^18^, 2% treatment, the highest dose showing no cell viability reduction, was selected as the optimal dose for HT-29 assays.

H_2_O_2_-induced oxidative stress triggers cell apoptosis. Therefore, the gene expression of H_2_O_2_-induced apoptosis markers was measured using RT-qPCR to determine the reactive oxygen species (ROS)-eliminating effects of F-SHWE (Fig. 2c–h). Compared to the cell positive control treated only with the cell growth medium, H_2_O_2_ treatment significantly upregulated the gene expression levels of *NF-κB*, *p53*, *BAX*, *cytochrome C*, *caspase 9*, and *caspase 3* (*p* < 0.05). However, *NF-κB* expression was downregulated 0.4-fold by SHWE compared with the H_2_O_2_-only treated group (*p* < 0.05) (Fig. 2c). Similarly, F-SHWE suppressed *NF-κB* expression by 0.9-fold, which was more than twice the inhibitory effect of SHWE (*p* < 0.05). Furthermore, F-SHWE normalized the gene expression of *p53*, *BAX*, *cytochrome C*, *caspase 9*, and *caspase 3*; however, SHWE did not affect these markers (*p* < 0.05) (Fig. 2d–h). These results suggest that F-SHWE can reduce oxidative stress-induced apoptosis. These effects were minimal with SHWE treatment, suggesting that fermentation enhanced the oxidative stress scavenging abilities.

### Effects of F-SHWE on LPS-induced inflammatory stress in HT-29 intestinal epithelial cells

**Figure 3:**
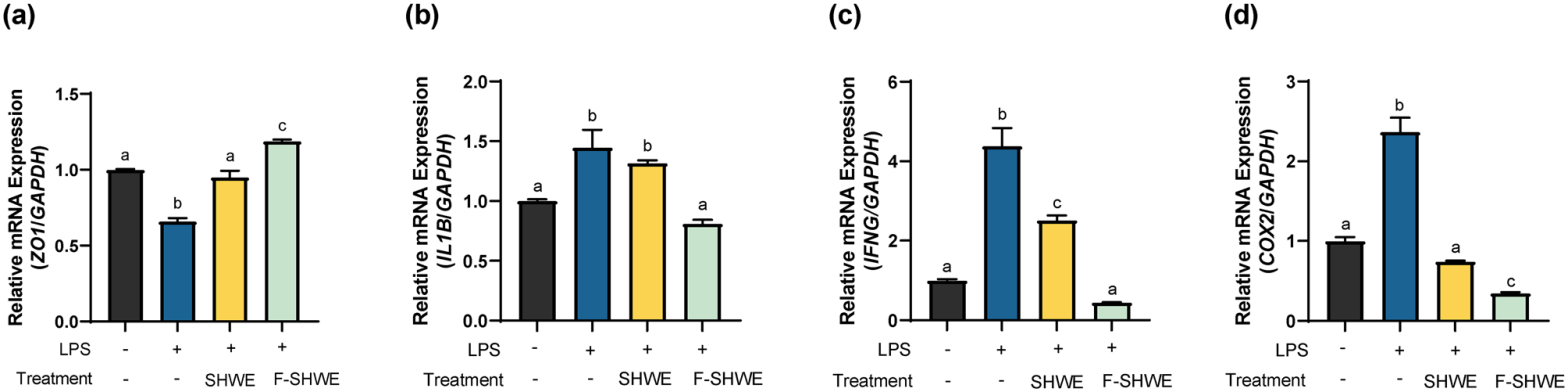
Effects of F-SHWE on LPS-induced stress in HT-29 cells. (**a**–**d**) Effects of SHWE or F-SHWE pre-treatment on the gene expression of inflammation-related markers in lipopolysaccharide-treated HT-29 cells. Results are expressed as mean ± SE (*n* = 3). ^abc^Results in the same series with different lowercase superscript letters are significantly different (*p* < 0.05; *IL1B*: *IL-1β, IFNG*: *IFN-γ*).

F-SHWE was used in an LPS-induced inflammatory cell model to determine its anti-inflammatory properties. The gene expression of inflammatory response-related markers *ZO1, IL1β, IFNγ*, and *COX2* was measured using RT-qPCR (Fig. 3a–d). LPS-induced inflammatory responses impaired gut barrier integrity by significantly reducing the expression of the tight junction protein, *ZO1*, leading to elevated *IL1β, IFNγ*, and *COX2* gene expression levels (*p* < 0.05) (Fig. 3a–d). SHWE and F-SHWE repaired *ZO1* expression; however, the effects were more pronounced in the F-SHWE treated group than in the SHWE group (*p* < 0.05) (Fig. 3a). Moreover, F-SHWE normalized the gene expression levels of *IL1β, IFNγ,* and *COX2*, downregulating them by at least 0.65-fold (*p* < 0.05) (Fig. 3b–d). SHWE did not affect *IL1β* expression; however, it inhibited *IFNγ*, although the effect was less pronounced than that of F-SHWE treatment (*p* < 0.05) (Fig. 3b–c). Compared with the LPS-only treated group, SHWE and F-SHWE exhibited a 1.87- and 3.93-fold reduction in *IFNγ* expression levels (*p* < 0.05) (Fig. 3c). Therefore, F-SHWE possesses potential anti-inflammatory effects, possibly enhanced through fermentation.

### Effects of F-SHWE on lipid accumulation and the expression of adipogenesis- and lipogenesis-related genes in differentiated 3T3-L1 adipocytes

**Figure 4:**
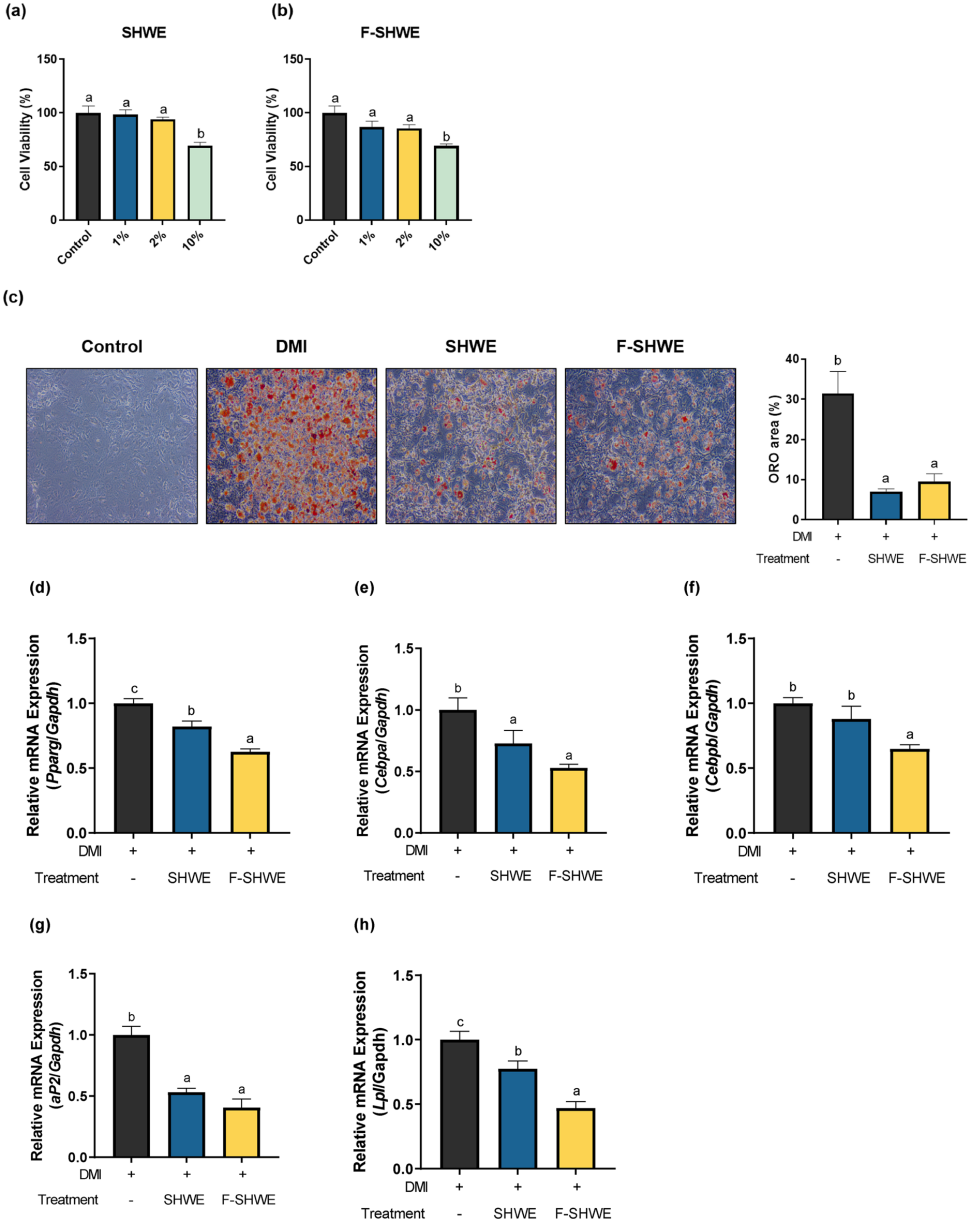
Effects of F-SHWE pre-treatment on adipogenesis in differential medium (DMI)-activated mature 3T3-L1 adipocytes. (**a**,**b**) Viability of 3T3-L1 cells pre-treated with different concentrations (0, 1, 2, 10%) of SHWE or F-SHWE for 24 h. (**c**) Oil red O staining images and stained area ratio of DMI-activated 3T3-L1 adipocytes pre-treated with SHWE or F-SHWE for 24 h. (**d**–**h**) Gene expression levels of adipocyte differentiation-related markers in DMI-activated 3T3-L1 adipocytes pre-treated with SHWE or F-SHWE for 24 h. Results are expressed as mean ± SE (*n* = 3). ^abc^Results in the same series with different lowercase superscript letters are significantly different (*p* < 0.05; ORO: Oil red O, *Pparg*: PPARγ, *Cebpa*: *C/EBPα*, *Cebpb*: *C/EBPβ*).

**Figure 5:**
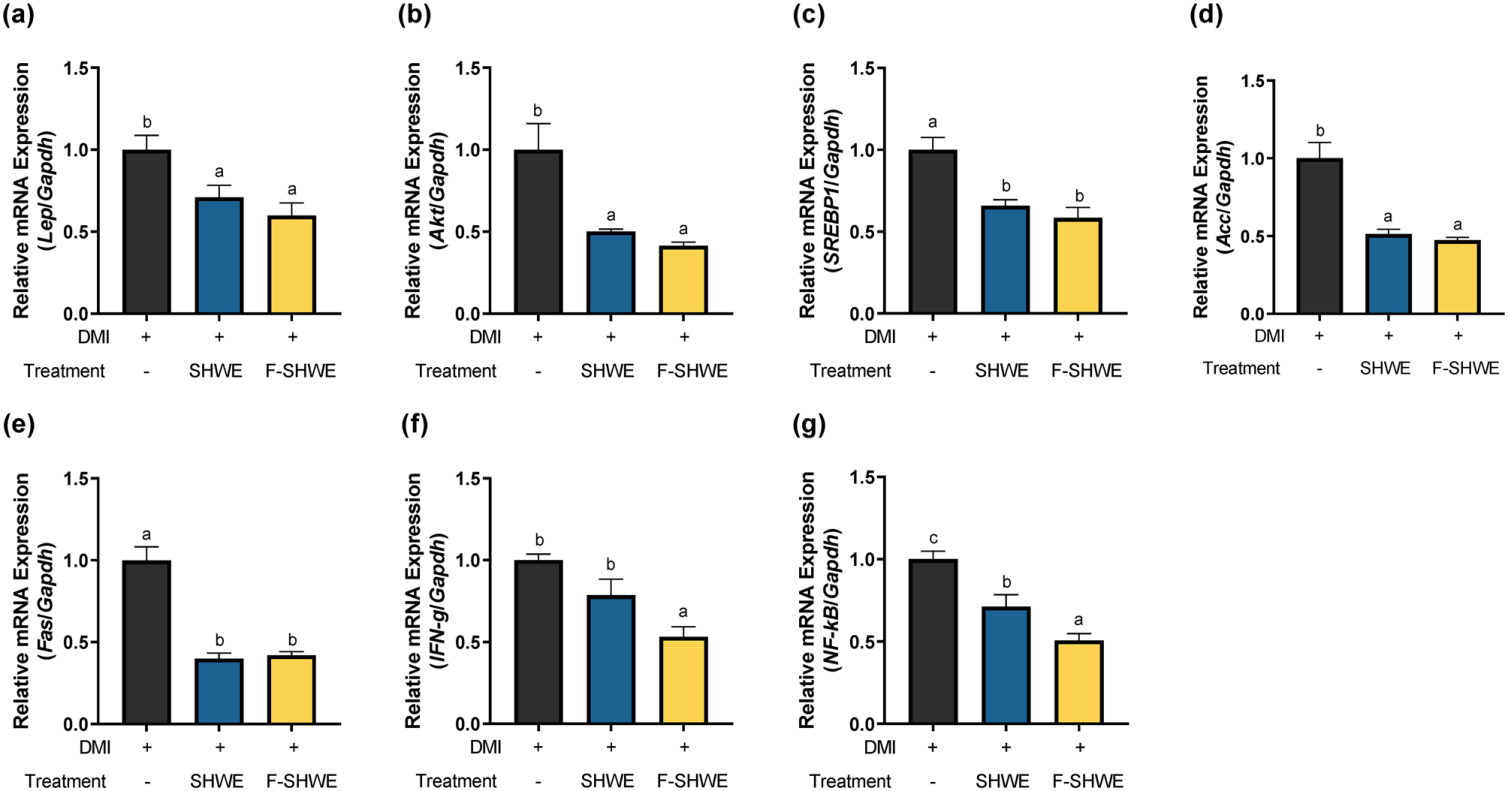
Effects of F-SHWE pre-treatment on the gene expression levels of lipogenesis and inflammatory cytokines in DMI-activated mature 3T3-L1 adipocytes. (**a**–**e**) Expression of adipogenesis-related genes in DMI-activated 3T3-L1 adipocytes pre-treated with SHWE or F-SHWE for 24 h. (**f**–**g**) Expression of inflammation-related genes in DMI-activated 3T3-L1 adipocytes pre-treated with SHWE or F-SHWE for 24 h. Results are expressed as mean ± SE (*n* = 3). ^abc^Results in the same series with different lowercase superscript letters are significantly different (*p* < 0.05; *Lep*: *Leptin*, *IFN-g*: *IFN-γ*).

Adipogenesis-related effects of F-SHWE were determined using Oil red O staining and RT-qPCR measurements of *PPARγ, C/EBPα, C/EBPβ, aP2,* and *Lpl* (Fig. 4). The optimal dosage for 3T3-L1 assays was selected based on the MTT results. At 10%, SHWE or F-SHWE reduced cell viability to 69%, which was cytotoxic (*p* < 0.05) (Fig. 4a–b). However, 1–2% SHWE or F-SHWE maintained cell viability above 85% (*p* < 0.05). Therefore, 2% was selected as the treatment dose for in vitro assays. DMI (a mixture of dexamethasone, IBMX, and insulin) induces preadipocyte differentiation, increasing lipid accumulation in differentiated mature adipocytes. SHWE and F-SHWE decreased DMI-induced lipid accumulation in mature adipocytes (Fig. 4c). Moreover, SHWE or F-SHWE downregulated DMI-induced gene expression of adipogenesis markers, *PPARγ*, *C/EBPα*, *aP2*, and *Lpl* (*p* < 0.05) (Fig. 4d–h). *C/EBPβ* gene expression was suppressed only by F-SHWE, suggesting the enhancing effect of fermentation on adipogenesis-related properties (*p* < 0.05) (Fig. 4f). Lipogenesis-related effects were determined by measuring *Leptin, Akt, SREBP1, Acc,* and *Fas* using RT-qPCR (Fig. 5a–e). SHWE or F-SHWE significantly downregulated all lipogenesis-related markers by at least 0.3-fold (*p* < 0.05). Moreover, F-SHWE downregulated the expression of inflammation markers (*IFN-γ* and *NF-κB*) by 0.5-fold (*p* < 0.05) (Fig. 5f–g). These results align with the suppressive effect of F-SHWE on *IFN-γ* and *NF-κB* expression in HT-29 cells (*p* < 0.05) (Figs. 2c and 3c). However, SHWE did not affect *IFN-γ* expression, while it reduced *NF-κB* expression by 0.29-fold (*p* < 0.05) (Fig. 5f–g). These results suggest that F-SHWE protected the adipocyte life cycle by reducing lipid accumulation, adipogenesis, lipogenesis, and inflammation in mature adipocytes.

### Effects of fermentation on the SCFA composition in F-SHWE

**Figure 6:**
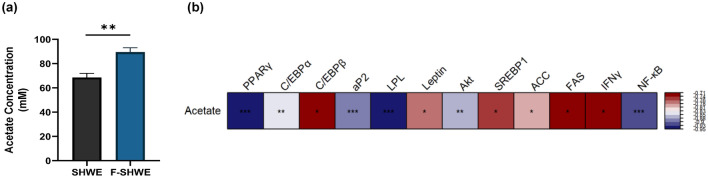
Effects of fermentation on acetate content in SHWE and F-SHWE (**a**) Acetate content in SHWE and F-SHWE. Results are expressed as mean ± SE (*n* = 3). Asterisks (*) indicate that acetate contents before and after fermentation differ significantly (*p < 0.05, **p < 0.01, ***p < 0.001). (**b**) Spearman correlation between gene expressions of 3T3-L1 markers and acetate level (*p < 0.05, **p < 0.01, ***p < 0.001).

GC/MS analysis revealed that SH803 fermentation increased the acetate content (Fig. 6a, Table 1). F-SHWE increased acetate content by 20.92 ± 2.89 mM (*p* < 0.05) compared with SHWE. Propionate, iso-butyrate, and butyrate were non-detected in both SHWE and F-SHWE (Table 1). For further confirmation on whether acetate is the key component of F-SHWE’s effects, spearman correlation analysis between gene expression levels of 3T3-L1 markers and acetate levels were carried out. The correlations of adipogenesis markers (*PPARγ, C/EBPα, aP2, Lpl*)*,* lipogenesis markers (*Akt*)*,* inflammation markers (*NF-κB*) were inversely proportional to acetate, indicating that these biomarkers may be involved in the protective effects of F-SHWE in 3T3-L1 cells (*p* < 0.05) (Fig. 6b). However, the correlation of *C/EBPβ, Leptin, Fas, SREBP1, ACC, IFN-γ* were proportional to acetate (*p* < 0.05), indicating the possibility of a different compound contributing to the effects of F-SHWE other than acetate. Overall, these results suggest that compositional change of acetate through fermentation may correlate with the adipocyte-protective effects of F-SHWE in 3T3-L1 cells.

**Table 1:** SCFA content before (SHWE) and after (F-SHWE) fermentation.

SCFA	SHWE (mM)	F-SHWE (mM)
Acetate	68.52 ± 3.42	89.44 ± 3.70**
Propionate	ND	ND
iso-Butyrate	ND	ND
Butyrate	ND	ND

Results are expressed as mean ± SE of triplicate runs (*n* = 3). Paired t-test analysis was performed with asterisks (*) indicating that SCFA content before and after fermentation differ significantly (*p < 0.05, **p < 0.01, ***p < 0.001, ND: non-detected).

## Discussion

ROS, a systemic physiological factor, facilitates adipogenic differentiation in 3T3-L1 pre-adipocytes, and as an outcome of differentiation, mature 3T3-L1 adipocytes end up with higher ROS levels than pre-adipocytes. Furthermore, excessive ROS accumulation induces cell death, such as apoptosis by activating NF-κB and p53 signaling, which triggers an apoptosis-inducing downstream signal of Bax, cytochrome C, caspase 9, and caspase 3^11,19^. In adipocytes, apoptosis worsens obesity by increasing macrophage infiltration into adipose tissue^20^. Therefore, recent studies attempt to attenuate obesity through antioxidative treatments^21,22^. Similarly, in this study, SHWE and F-SHWE both possessed oxygen radical scavenging effects comparable to ascorbic acid, a commercially used antioxidant (Fig. 1f). On treating intestinal epithelial cells, SHWE suppressed apoptosis by reducing *NF-κB* expression; F-SHWE had a greater suppressive effect than SHWE by downregulating *NF-κB* and *p53* expression, indicating that SHWE and F-SHWE have anti-obesity potential (Fig. 2).

Inflammation is also associated with adipogenesis. Long term consumption of a high-fat diet, a common risk factor for obesity, induces systemic chronic low-grade inflammation^23^. This in turn alters gut microbiota composition and damages intestinal barrier integrity, increasing endotoxin levels in the intestinal lumen and plasma ^24,25^. Endotoxins such as LPS intensify the damage of gut barrier proteins (such as ZO1) and induce TLR4 signaling ^24^, consequently promoting the production of pro-inflammatory cytokines (such as IL1β), which exacerbate systemic low-grade inflammation and accelerate obesity pathogenesis ^26–28^. Moreover, LPS can increase the expression of cyclooxygenase-2 (COX2)—a pro-inflammatory enzyme that stimulates prostaglandin production^29,30^. In this study, SHWE fermentation improved the protective effects against LPS-induced intestinal inflammation by increasing tight junction protein and reducing pro-inflammatory cytokine gene expression levels (Fig. 3). Notably, *COX2* expression was reduced by F-SHWE treatment. Besides its pro-inflammatory properties, COX2 can inhibit adipogenesis by downregulating PPARγ and C/EBPα expression, thereby suppressing NF-κB signaling cascades, which promote pro-inflammatory cytokine expression (IFN-γ) ^31^. While reduced *COX2* expression by F-SHWE indicates anti-inflammatory efficacy, there is potential for reduced anti-adipogenic effects as well. Therefore, for the assessment of adipogenesis-related efficacy, further evaluations using an adipocyte differentiation model are necessary. Conventionally, 3T3-L1 murine preadipocytes are used as in vitro adipogenesis models; they enable preadipocyte differentiation pathway-related analysis^32^. Moreover, 3T3-L1 preadipocytes are differentiated by culturing in a growth medium treated with a mixture of IBMX, dexamethasone, and insulin. Dexamethasone-induced C/EBPδ and IBMX-induced C/EBPβ heterodimerize to activate PPARγ and C/EBPα, which are known to promote preadipocyte maturation into functional adipocytes^32,33^. Additionally, PPARγ and C/EBPα regulate the activation of mature adipocyte markers, such as aP2 and LPL^33^. These signaling cascades lead to lipid accumulation in adipocytes, which can be measured using Oil Red O staining^34^. As shown in Fig. 4, SHWE and F-SHWE treatment reduced Oil Red O stainable lipid accumulation in 3T3-L1 cells and the expression of adipogenesis markers (*PPARγ, C/EBPα, aP2,* and *Lpl*). These results align with those for the inhibitory effects of SHWE and F-SHWE on lipogenesis-related gene expression (Fig. 5). Lipogenesis is the synthesis of lipids within mature adipocytes, activated by the downstream signaling of Akt, SREBP1, ACC, and Fas^35,36^. Overall, these results indicate that SHWE and F-SHWE have the potential as anti-obesity agents by reducing adipogenesis and lipogenesis. Despite the higher inhibition rate of ROS-mediated apoptosis by F-SHWE than that by SHWE, both these extracts exhibited similar adipogenesis and lipogenesis downregulation. However, F-SHWE was more effective than SHWE in lowering inflammation response marker expressions in adipocytes; inflammatory cytokine *IFN-γ* and transcription factor *NF-κB* (Fig. 5f and g). IFN-γ impairs mitochondrial function and fatty acid flux in adipocytes, accelerating inflammation through responses, such as NF-κB regulated pathways^37^. Interestingly, in a previous report, *Lactobacillus*-fermented *Sargassum* spp. inhibited NF-κB signaling and increased SCFA productions^38^. SCFA, organic monocarboxylic acids with less than six carbons, reduce systemic inflammation through decreased NF-κB signaling and pro-inflammatory cytokines in obese states^39^. Thus, amounts of major SCFA such as acetate (C2), propionate (C3), and butyrate (C4), were measured to assess the effect of fermentation on its production. As shown in Fig. 6a, acetate concentrations were higher in F-SHWE than SHWE. Other SCFA were non-detected in both SHWE and F-SHWE. Acetate is produced by LAB strains through the subsequent conversion of pyruvate to acetyl phosphate and then to acetate in the presence of acetyl-CoA^40^. In a previous report, acetate reduced lipid accumulation in 3T3-L1 adipocytes through attenuation of fatty acid oxidation^41^. Similarly, in our study, lipid accumulation was reduced in F-SHWE treatment, which had higher concentrations of acetate than that of SHWE (Fig. 4c). Moreover, correlation analysis revealed inverse relationships between acetate concentrations and gene expressions of adipogenesis markers (*PPARγ, C/EBPα, aP2, Lpl*)*,* lipogenesis markers (*Akt*)*,* and inflammation markers (*NF-κB*) (Fig. 6b). This indicates acetate as a potent key contributor to the anti-obesity effects of F-SHWE in 3T3-L1 cells.

This study had some limitations that warrant discussion. First, only in vitro experiments were conducted to examine the health benefits of F-SHWE. Oxidation-, inflammation-, and adipocyte metabolism-related effects were notable in adipocytes and intestinal cells treated with F-SHWE. However, these results cannot represent the possible interactions between the intestine and adipocytes on consuming food supplements. To further validate these effects and the related mechanisms of F-SHWE on oral consumption, in vivo studies capable of examining gut-adipose tissue signaling via pathways such as the gut microbiome, adipokines, and inflammatory cytokines are necessary. Second, to confirm whether acetate is the key component responsible for the fermentation-induced aspects of F-SHWE, the effects of F-SHWE and the equivalent amount of acetate should be compared in the same experimental models used in this study. Lastly, metabolites other than acetate may contribute to the fermentation-induced characteristics of F-SHWE. Correlations of *C/EBPβ, Lep, Fas, SREBP1, Acc, Fas, IFN-γ* gene expression were proportional to acetate, indicating the possibility of different compounds other than acetate also participating in the protective activities of F-SHWE. Other major metabolites produced by LAB-fermentation such as lactic acid could be potential functional compounds in F-SHWE. Lactic acid form acidic conditions in the intestine that result in inhibitory effects on obesity-related pathogenic bacteria^42^. In a previous report, *Lactobacillus*-fermentation of seaweed species was effective in lactic acid production^43^, indicating the possibility of it as a bioactive component for F-SHWE. Thus, the presence of these molecules may contribute to the effects of F-SHWE on adipocytes. Therefore, additional studies on the effects of F-SHWE oral supplementation in an animal model and on F-SHWE compositional analysis are in progress.

In conclusion, *S. horneri* fermented by *Lactiplantibacillus pentosus* SH803 inhibited adipogenesis and lipid accumulation induced by 3T3-L1 preadipocyte differentiation. The bioactive compounds eliminated apoptosis-induced oxidative (ROS) and inflammatory (LPS) stress in intestinal cells, exhibiting promising potential as functional food additives. These antioxidative and anti-inflammatory effects of F-SHWE may have contributed to adipogenesis reduction through PPARγ-mediated signaling and lipogenesis reduction through Akt-mediated Fas signaling. Moreover, F-SHWE inhibited inflammation in adipocytes by suppressing IFNγ and NF-κB expression. Compositional analysis revealed that fermentation increased acetate levels, which may have contributed to the enhanced properties in F-SHWE. Further validation studies are needed using in vivo models and are in progress. Overall, employing *S. horneri* fermentation as a therapeutic anti-obesity agent is a promising strategy to utilize this common but invasive species of the coastal ecosystem.

## Materials and methods

### Materials

*S. horneri* samples were collected along the shores of Jeju Island, South Korea. LAB strains, *Lactiplantibacillus pentosus* SH803 (100% identity, accession no. NR_029133.1), *Lactobacillus plantarum* HJ617 (99.8% identity, accession no. NR_115605.1), *Lactobacillus acidophilus* SJ422 (100% identity, accession no. NR_113638.1), and *Lactobacillus acidophilus* SH123 (99.86% identity, accession no. NR_117062.1), were obtained from the Food Microbiology Laboratory at Korea University (Seoul, South Korea). Additionally, 16S rRNA sequencing was performed by Macrogen (Seoul, South Korea) for strain identification. Sequence datasets generated during and/or analyzed during the current study are available in the GeneBank (NCBI-Nucleotide Database) repository and summarized in Supplementary Table ST. 1. All strains were grown in De Man, Rogosa, and Sharpe (MRS) broth (Kisan Bio, Seoul, South Korea) at 37 °C for 18 h and subcultured thrice before use.

### Preparation of LAB-fermented *S. horneri*

S. horneri water extracts and fermented products of those were prepared^44^. *S. horneri* was washed, dried at 50 °C for 24 h, and ground. The resulting powder was mixed with distilled water (1:50 w/v) and autoclaved (121 °C, 15 min) for sterilization. After cooling, cellulase (1:50 v/v, Sigma-Aldrich, MO, USA) was added, incubated (50 °C, pH 4.5) for 48 h, and autoclaved (121 °C, 15 min) for cellulase denaturation and sample sterilization^44^. The resulting mixture was termed *S. horneri* water extract (SHWE). Subsequently, the minimal broth (peptone [1:100 w/v], sodium acetate 3H_2_O [1:200 w/v], magnesium sulfate 7H_2_O [1:10,000 w/v], manganese sulfate 4H_2_O [1:20,0000 w/v], 5 mL tween 80 [1:100 v/v], diammonium citrate [1:500 w/v], dipotassium phosphate [1:500 w/v], and distilled water adjusted to a total volume of 500 mL) was mixed with SHWE and used as bacterial strain culture medium. Thereafter, SH803 culture was centrifuged at 10,800 × g for 3 min (VS-180Cfi, Vision Scientific Co., Daejeon, Korea) and washed twice with phosphate-buffered saline (PBS). Next, the optical density of the harvested bacterial pellets at 600 nm was adjusted to 0.3; the pellets were added to *S. horneri* culture medium (1:100 v/v) and incubated for 48 h. Sample preparations were done at 0 h and 48 h fermentation and spread on MRS agar plates (Kisan Bio) to assess bacterial growth. SHWE fermented with SH803 was termed F-SHWE. Subsequently, F-SHWE was filtered using 0.45 μm Stericup filters (Merck, Darmstadt, Germany), freeze dried, and kept at − 80 °C until use.

### Antioxidative activity evaluation using DPPH assay

Investigation of antioxidant activity was measured by the DPPH method, with slight modifications^45^. DPPH (2,2-diphenyl-1-picrylhydrazyl) solutions were prepared by dissolving 0.4 mM DPPH (Sigma-Aldrich) in ethanol (Duksan Chemicals, Incheon, South Korea) until the absorbance at 517 nm was 0.94–0.97. In addition, ascorbic acid solutions were prepared dose-dependently (0.6, 1.2, and 2.4 mM) and used as positive controls. Next, the samples (60 μL) were mixed with 1000 μL DPPH solution and kept in complete darkness for 30 min at room temperature (25 °C). After incubation, absorbance was determined at 517 nm using the Epoch microplate spectrophotometer (BioTek, VT, USA). DPPH radical scavenging activity was calculated using the following equation.

$$ {\text{DPPH radical scavenging activity }}\left( \% \right) \, = \, \left( {{1} - \left[ {{\text{Abs}}_{{{\text{sample}}}} } \right]/\left[ {{\text{Abs}}_{{{\text{blank}}}} } \right]} \right) \, \times {1}00 $$

where Abs_sample_ is the absorbance of the sample mixed with DPPH solution, and Abs_blank_ is the absorbance of the sample solvent mixed with DPPH solution.

### Cell culture

HT-29 human colorectal adenocarcinoma cells were obtained from the Korean Cell Line Bank (KCTC, Seoul, South Korea). The cells were maintained in culture dishes containing Roswell Park Institute 1640 Medium (RPMI, Gibco, Dublin, Ireland) with 10% fetal bovine serum (FBS, Hyclone, MA, USA) and 1% penicillin/streptomycin (P/S, GE Healthcare, Chicago, IL, USA). Furthermore, 3T3-L1 murine preadipocytes were also obtained from KCTC. The cells were maintained in cell culture dishes containing Dulbecco’s Modified Eagle Medium (DMEM), low glucose (Gibco, Dublin, Ireland) with 10% bovine calf serum (Hyclone, MA, USA), and 1% P/S (GE Healthcare). Both the cell types were incubated in a humid atmosphere (37 °C, 5% CO_2_).

### Cell cytotoxicity assessment using the MTT assay

Cell cytotoxicity was measured by the MTT assay with slight modifications^45^. MTT (3-(4,5-dimethylthiazol-2-yl)-2,5-diphenyl-2H-tetrazolium bromide) assay was performed to assess the toxicity of F-SHWE on HT-29 and 3T3-L1 cells. The cells were seeded at a density of 5 × 10^5^ cells/well in 96-well plates. After 24 h incubation, the cells were pre-treated with F-SHWE dose-dependently (0.2, 1, 2, and 10%) and incubated for 24 h (37 °C, 5% CO_2_). Subsequently, the cells were treated with MTT solution (5 mg/mL, Sigma-Aldrich) and incubated for 1 h. After media removal, the plates were washed with PBS and treated with dimethyl sulfoxide (Sigma-Aldrich). Eventually, absorbance was measured at 540 nm using the Epoch microplate spectrophotometer (BioTek, VT, USA), and the relative percentage of cell proliferation was calculated.

### Gene expression measurement using reverse transcription quantitative real-time polymerase chain reaction (RT-qPCR)

Gene expressions of biomarkers were measured using RT-qPCR^46^. For antioxidative activity evaluation, cells were seeded at a density of 5 × 10^5^ cells/well in 12-well plates. After 24 h incubation, the cells were pre-treated with F-SHWE (1:2 v/v, diluted with RPMI) and incubated for 24 h (37 °C, 5% CO_2_). To induce oxidative stress, the cells were treated with 100 μM hydrogen peroxide (H_2_O_2,_ Duksan, Ansan, South Korea) diluted with RPMI and incubated for 24 h (37 °C, 5% CO_2_). Next, total mRNA was extracted using TRIzol reagent (Life Technologies, CA, USA) following the manufacturer’s instructions. The concentration and purity of the extracted RNA were assessed using the NanoDrop spectrophotometer (BioTek, VT, USA) and standardized to a final concentration of 0.1 μg/μL. Following this, cDNA was synthesized using the reverse transcription kit (Thermo- Fisher). The polymerase chain reaction (PCR) cycling conditions were 25 °C for 10 min, 37 °C for 120 min, and 85 °C for 5 min. Subsequently, reverse transcription quantitative real-time PCR (RT-qPCR) was performed using the Bio-Rad CFX96 Real-Time PCR Detection System (Bio-rad, Hercules, CA, USA). Targeted genes were quantified using MG2 x qPCR MasterMix (SYBR green) (MGmed, South Korea). The RT-qPCR cycling conditions were: an initial denaturation cycle at 95 °C for 10 min, followed by 40 cycles of amplification at 95 °C for 15 s, annealing at 55–65 °C for 30 s, and extension at 70 °C for 5 s. Lastly, the mRNA expression levels of each targeted gene were analyzed and normalized to the internal standard gene, *GAPDH*, using Bio-Rad CFX Maestro (Bio-Rad Laboratories, Hercules, CA, USA). The primer sequences used in this study are listed in Supplementary Table ST. 2.

For anti-inflammatory activity evaluation, cells were seeded at a density of 5 × 10^5^ cells/well in 12-well plates. After 24 h incubation, the cells were pre-treated with F-SHWE (1:2 v/v, diluted with RPMI) and incubated for 24 h (37 °C, 5% CO_2_). To stimulate inflammatory responses, 1 μg/mL lipopolysaccharide (LPS, Sigma-Aldrich) diluted with RPMI was added and incubated for 24 h (37 °C, 5% CO_2_). Subsequent procedures were similar to those performed for evaluating antioxidative activity using RT-qPCR.

For evaluation of adipocyte differentiation-related gene expression levels, 3T3-L1 cells were treated with post-differentiation media as described in Section “Gas Chromatography/Mass Spectrometry (GC/MS) instrumentation and chromatographic condition” and incubated for 6 days. The media was replaced every 2 days. Following this, the total mRNA was extracted using TRIzol reagent (Life) according to the manufacturer’s instructions. The RNA, PCR, and RT-qPCR analyses were performed as described in section “Gene expression measurement using reverse transcription quantitative real-time polymerase chain reaction (RT-qPCR)”. The primer sequences used in this study are listed in Supplementary Table ST. 3.

### Lipid accumulation assessment by Oil Red O Staining

Lipid accumulation was evaluated with Oil Red O staining^47^. Initially, 3T3-L1 cells were seeded at a density of 0.8 × 10^5^ cells/well in six-well plates and incubated for 48 h. After incubation, the cell culture media was replaced and incubated for 72 h. Further, cells were co-treated with differentiation medium (DMI; 0.5 mM 3-isobutyl-1-methylanthine [IBMX], 1 μM dexamethasone, 5 μg/mL insulin, 10% FBS, and 1% P/S mixed in DMEM, low glucose) and F-SHWE (1:2 v/v, diluted with differentiation medium) and incubated for 48 h. Subsequently, the cells were treated with post-differentiation media (5 μg/mL insulin, 10% FBS, and 1% P/S mixed in DMEM, low glucose) and incubated for 6 days; the media was replaced every 2 days. After the 6-day incubation, cells were fixed with formaldehyde (10% v/v) for 10 min and washed with isopropanol (60% v/v). Subsequently, 1 mL Oil Red O staining solution (0.5% v/v in isopropanol, Sigma-Aldrich) was added to the cells and incubated at room temperature for 20 min. Finally, the cells were washed with PBS and observed using the Olympus CKX41 inverted phase contrast microscope (Olympus, Tokyo, Japan). Oil Red O-stained area ratio (%) was measured using Image J software (National Institutes of Health, MA, USA).

### Gas chromatography/mass spectrometry (GC/MS) instrumentation and chromatographic condition

Short chain fatty acid (SCFA) contents of SHWE and F-SHWE were determined using GC/MS, per the method described by Eor et al. with slight modifications^48^. Briefly, 50μL of SHWE or F-SHWE samples were combined with 100 µL crotonic acid, 50 µL HCl, and 200 µL ether, and then homogenized and centrifuged at 1000 ×*g* for 10 min. Supernatants were transferred to vials and 16 µL N-tert-butyldimethylsilyl-N-methylttrifluoroacetamide (Sigma‒Aldrich, USA) was added. After mixing, vials were sealed and heated at 80 ℃ for 20 min, and then kept in room temperature for 48 h. Samples were placed in a 6890N Network GC System (Agilent Technologies, California, USA) with a HP-5MS column (30 m, 0.25 mm, 0.25 µm) and 5973 Network Mass Selective Detector (Agilent Technologies, USA). Helium (99.9999% purity) was used as a delivery gas at a flow rate of 1.2 mL/min. The head pressure was 97 kPa and the split was 20:1. The inlet and transfer line temperatures were 250 and 260 ℃, respectively. The following temperature program was used: 60 ℃ (3 min), 60–120 ℃ (5 ℃ min), 120–300 ℃ (20 ℃ min). One microliter of sample was injected with 30 min of run time. SCFA concentrations were qualified by comparing their peak areas with those of the standards.

### Statistical analyses

Statistical analyses were performed using IBM SPSS Statistics software version 25.0 (IBM, Armonk, NY, USA). One-way analysis of variance with Tukey’s test was used to analyze the statistical difference between the mean values of samples. Statistical significance was set at *p* < 0.05. All figures were drawn using GraphPad Prism 9.0 (GraphPad Software, Boston, MA, USA). Correlation based analyses and visualization were performed using R studio (RStudio, United States) and related packages.

### Supplementary Information

Supplementary Information.

## Data Availability

Sequence datasets generated during and/or analyzed during the current study are available in the GeneBank (NCBI-Nucleotide Database) repository as follows and summarized in Supplementary Table ST. 1. 1. *Lactiplantibacillus pentosus* strain SH803 16 s ribosomal RNA gene, partial sequence under accession NR_029133.1 (link: https://www.ncbi.nlm.nih.gov/nuccore/NR_029133.1). 2. *Lactiplantibacillus plantarum* strain HJ617 16 s ribosomal RNA gene, partial sequence under accession number NR_115605.1 (link: https://www.ncbi.nlm.nih.gov/nuccore/NR_115605.1). 3. *Lactobacillus acidophilus* strain SJ422 16 s ribosomal RNA gene, partial sequence under accession number NR_113638.1 (link: https://www.ncbi.nlm.nih.gov/nuccore/NR_113638.1). 4. *Lactobacillus acidophilus* strain SH123 16 s ribosomal RNA gene, partial sequence under accession number NR_117062.1 (link: https://www.ncbi.nlm.nih.gov/nuccore/NR_117062.1).
