# Supplementary material for: Sargassum horneri extract fermented by Lactiplantibacillus pentosus SH803 mediates adipocyte metabolism in 3T3-L1 preadipocytes by regulating oxidative damage and inflammation
Source: Sci Rep. 2024 Jul 2;14:15064. doi: 10.1038/s41598-024-65956-8 (PMC11220060; doi:10.1038/s41598-024-65956-8)
Supplement: Supplementary file 1 — Supplementary Information. [file 41598_2024_65956_MOESM1_ESM.pdf]

***Sargassum horneri* extract fermented by *Lactiplantibacillus pentosus* SH803 mediates adipocyte metabolism in 3T3-L1 preadipocytes by regulating oxidative damage and inflammation**

Jae-Young Kim[1,2], Sejin Jang[1], Hyun Ji Song[1], SangHoon Lee[1], Sejin Cheon[1], Eun Jin Seo[1], Yi Hyun Choi[1], and Sae Hun Kim[1,2*]

[1]College of Life Sciences and Biotechnology, Korea University, Seoul 02841, Republic of Korea

[2]Institute of Life Science and Natural Resources, Korea University, Seoul 02841, Republic of Korea

Author ORCIDs: Jae-Young Kim (0000-0003-1937-9535), Sejin Jang (0009-0003-6781-1473), Hyun Ji Song (0009-0005-6629-7817), SangHoon Lee (0009-0000-4216-3949), Sejin Cheon (0009-0007-0298-7450), Eun Jin Seo (0009-0003-8540-1400), Yi Hyun Choi (0009-0006-0513-1503), Sae Hun Kim (0000-0002-0990-2268)

[*]**Corresponding authors:**

Dr. Sae Hun Kim, Ph.D.

Email: saehkim@korea.ac.kr

Supplementary Table ST. 1. Identification of bacterial strains based on 16S rRNA gene sequencing data

Strain - ID	Source of isolation	Strain name/Genus species	16S rRNA gene (number of nucleotides)	Accession number	Similarity of 16S rRNA gene sequence	
					Match/Total	Percent (%)
SH803	*Kimchi*	*Lactiplantibacillus pentosus*	1519	NR_029133.1	1493/1493	100
HJ617	*Kimchi*	*Lactiplantibacillus plantarum*	1519	NR_115605.1	1500/1503	99.8
SJ422	*Kimchi*	*Lactobacillus acidophilus*	1489	NR_113638.1	1489/1489	100
SH123	*Kimchi*	*Lactobacillus acidophilus*	1531	NR_117062.1	1504/1506	99.86

Supplementary Table ST. 2. HT-29 primer sequences used in reverse transcription quantitative real-time polymerase chain reaction (RT-qPCR) analysis

	Gene	Sequence	Tm (°C)
Apoptosis-related genes	*NF-κB*	F: 5′- AAC AGA GAG GAT TTC GTT TCC G -3′<br>R: 5′- TTT GAC CTG AGG GTA AGA CTT CT -3′	62.4
	p53	F: 5′- GAG GTT GGC TCT GAC TGT ACC -3′<br>R: 5′- TCC GTC CCA GTA GAT TAC CAC -3′	63.3
	BAX	F: 5′- CCC GAG AGG TCT TTT TCC GAG -3′<br>R: 5′- CCA GCC CAT GAT GGT TCT GAT -3′	63.3
	Cytochrome C	F: 5′- CTT TGG GCG GAA GAC AGG TC -3′<br>R: 5′- TTA TTG GCG GCT GTG TAA GAG -3′	64.5
	Caspase 9	F: 5′- CTC AGA CCA GAG ATT CGC AAA C -3′<br>R: 5′- GCA TTT CCC CTC AAA CTC TCA A -3′	59.0
	Caspase 3	F: 5′- CAT GGA AGC GAA TCA ATG GAC T -3′<br>R: 5′- CTG TAC CAG ACC GAG ATG TCA -3′	61.4
Inflammation-related genes	*ZO1*	F: 5′- GAG GCC AAG CCC TGG TAT G -3′<br>R: 5′- CGG GCC GAT TGA TCT CAG C -3′	62.1
	IL1B	F: 5′- ACT GAG AGT GAT TGA GAG TGG AC -3′<br>R: 5′- AAC CCT CTG CAC CCA GTT TTC -3′	61.0
	IFNG	F: 5′- TCG GTA ACT GAC TTG AAT GTC CA -3′<br>R: 5′- TCG CTT CCC TGT TTT AGC TGC -3′	62.4
	COX2	F: 5′- GAA TCA TTC ACC AGG CAA ATT G -3′<br>R: 5′- TCT GTA CTG CGG GTG GAA CA -3′	60.9
Housekeeping gene	*GAPDH*	F: 5′- CCT GCT TCA CCA CCT TCT TG -3′<br>R: 5′- ATG ACC ACA GTC CAT GCC ATC -3′	60.8

Supplementary Table ST. 3. 3T3-L1 primer sequences used in RT-qPCR analysis

	Gene	Sequence	Tm (°C)
Adipogenesis-related genes	*Pparg*	F: 5′- GGA AGA CCA CTC GCA TTC CTT -3′<br>R: 5′- GTA ATCA GCA ACC ATT GGG TCA -3′	59.0
	Cebpa	F: 5′- CAA GAA CAG CAA CGA GTA CCG -3′<br>R: 5′- GTC ACT GGT CAA CTC CAG CAC -3′	59.0
	Cebpb	F: 5′- GCA AGA GCC GCG ACA AG -3′<br>R: 5′- GGC TCG GGC AGC TGC TT -3′<br>R: 5′- CAT AGG GGG CGT CAA ACA G -3′	59.0
	aP2	F: 5′- AAG GTG AAG AGC ATC ATA ACC CT -3′<br>R: 5′- TCA CGC CTT TCA TAA CAC ATT CC -3′	55.7
	Lpl	F: 5′- GGG AGT TTG GCT CCA GAG TTT -3′<br>R: 5′- TGT GTC TTC AGG GGT CCT TAG -3′	55.7
Lipogenesis-related genes	*Lep*	F: 5′- TTC ACA CAC GCA GTC GGT ATC -3′<br>R: 5′- GGC TGG TGA GGA CCT GTT G -3′	63.3
	Akt	F: 5′- ATG AAC GAC GTA GCC ATT GTG -3′<br>R: 5′- TTG TAG CCA ATA AAG GTG CCA T -3′	55.7
	SREBP1	F: 5′- GAT GTG CGA ACT GGA CAC AG -3′<br>R: 5′- CAT AGG GGG CGT CAA ACA G -3′	57.0
	Acc	F: 5′- GGA CCA CTG CAT GGA ATG TTA A -3′<br>R: 5′- TGA GTG ACT GCC GAA ACA TCT C -3′	55.7
	Fas	F: 5′- TAT CAA GGA GGC CCA TTT TGC -3′<br>R: 5′- TGT TTC CAC TTC TAA ACC ATG CT -3′	61.4
Inflammatory cytokines	*IFN-g*	F: 5′- ACA GCA AGG CGA AAA AGG ATG -3′<br>R: 5′- TGG TGG ACC ACT CGG ATG A -3′	55.7
	NF-κB	F: 5′- GGA GGC ATG TTC GGT AGT GG -3′<br>R: 5′- CCC TGC GTT GGA TTT CGT G -3′	55.7
Housekeeping gene	*Gapdh*	F: 5′- AGG TCG GTG TGA ACG GAT TTG -3′<br>R: 5′- GGG GTC GTT GAT GGC AAC A -3′	59.0
